# Radiolabeled novel mAb 4G1 for immunoSPECT imaging of EGFRvIII expression in preclinical glioblastoma xenografts

**DOI:** 10.18632/oncotarget.14088

**Published:** 2016-12-22

**Authors:** Xujie Liu, Chengyan Dong, Jiyun Shi, Teng Ma, Zhongxia Jin, Bing Jia, Zhaofei Liu, Li Shen, Fan Wang

**Affiliations:** ^1^ Medical Isotopes Research Center and Department of Radiation Medicine, School of Basic Medical Sciences, Peking University, Beijing 100191, China; ^2^ Department of Cell Biology, School of Basic Medical Sciences, Peking University, Beijing 100191, China; ^3^ Key Laboratory of Protein and Peptide Pharmaceuticals, CAS Center for Excellence in Biomacromolecules, Institute of Biophysics, Chinese Academy of Sciences, Beijing 100101, China

**Keywords:** EGFRvIII, monoclonal antibody, specificity, tumor, SPECT imaging

## Abstract

Epidermal growth factor receptor mutant III (EGFRvIII) is exclusively expressed in tumors, such as glioblastoma, breast cancer and hepatocellular carcinoma, but never in normal organs. Increasing evidence suggests that EGFRvIII has clinical significance in glioblastoma prognosis due to its enhanced tumorigenicity and chemo/radio resistance, thus the development of an imaging approach to early detect EGFRvIII expression with high specificity is urgently needed. To illustrate this point, we developed a novel anti-EGFRvIII monoclonal antibody 4G1 through mouse immunization, cell fusion and hybridoma screening and then confirmed its specificity and affinity by a serial of assays. Following biodistribution and small animal single-photon emission computed tomography (SPECT/CT) imaging of ^125^I-4G1 in EGFRvIII positive/negative tumor-bearing mice were performed and evaluated to verify the tumor accumulation of this radiotracer. The biodistribution indicated that ^125^I-4G1 showed prominent tumor accumulation at 24 h post-injection, which reached maximums of 11.20 ± 0.75% ID/g and 13.98 ± 0.57% ID/g in F98npEGFRvIII and U87vIII xenografts, respectively. In contrast, ^125^I-4G1 had lower tumor accumulation in F98npEGFR and U87MG xenografts. Small animal SPECT/CT imaging revealed that ^125^I-4G1 had a higher tumor uptake in EGFRvIII-positive tumors than that in EGFRvIII-negative tumors. This study demonstrates that radiolabeled 4G1 can serve as a valid probe for the imaging of EGFRvIII expression, and would be valuable into the clinical translation for the diagnosis, prognosis, guiding therapy, and therapeutic efficacy evaluation of tumors.

## INTRODUCTION

The epidermal growth factor receptor (EGFR), a member of the ErbB family of receptor tyrosine kinases, is a 170 kDa transmembrane glycoprotein that is overexpressed in a variety of solid tumors [1–3]. Multiple ligands, including EGF, transforming growth factor-a, amphiregulin, betacellulin, and epiregulin, activate EGFR. Upon binding of the ligand to the EGFR extracellular domain, downstream pathways and several downstream signaling cascades are activated to induce the transcription of genes involved in cell proliferation, tumor invasion, and metastasis [4]. A variety of EGFR-targeting chemotherapeutic drugs, such as tyrosine kinase inhibitors (TKIs), and monoclonal antibodies (mAbs) have been developed to treat cancer patients by blocking EGFR signal transduction [5, 6]. However, a large number of studies show that only a minority of patients benefit from these drugs, and the limited efficacy of small molecule TKIs (gefitinib and erlotinib) and mAbs (cetuximab, panitumumab, and zalutumumab) against EGFR is mainly attributed to EGFR mutations [7–9].

Among EGFR mutations, EGFR variant III (EGFRvIII) has attracted increasing attention. EGFRvIII harbors an in-frame deletion of exons 2-7 that encode amino acid residues 6-273. This deletion produces a truncated 150 kDa protein that shows weak but constitutive oncogenic activation in a ligand-independent manner [10–12]. Glioblastoma multiforme, the most common and malignant type of primary brain tumors, is often associated with EGFRvIII expression [13, 14]. Such expression can be regarded as a marker of poor prognosis because of its potential to confer enhanced tumorigenicity to gliomas [15–17]. Recent studies have demonstrated that EGFRvIII is expressed in glioma stem cells (GSCs) and may be a biomarker of GSCs [18]. Moreover, increasing studies have confirmed that EGFRvIII-positive cells are more radioresistant and chemoresistant to EGFR inhibitors because of the constitutively active receptor tyrosine kinase in EGFRvIII [19–21]. Thus, it is necessary to examine EGFRvIII expression before and after radio/chemotherapy for patient prognosis and prediction of drug resistance to administer the appropriate individualized therapy. Thus far, several EGFRvIII detection methods have been developed, including reverse transcription-polymerase chain reaction (RT-PCR), real-time RT-PCR, tumor immunohistochemistry, and blood tests. However, these detection methods usually result in inconsistent results due to the limitations of their methodology [22–24]. More importantly, these methods are not applicable to noninvasive *in vivo* detection or real-time monitoring of EGFRvIII expression.

In recent years, molecular imaging has emerged as a novel and rapidly growing multidisciplinary research field with the combination of molecular biology and *in vivo* imaging [25]. Molecular imaging not only enables noninvasive *in vivo* imaging, which reflects biological processes at cellular and sub-cellular levels, but also allows real-time monitoring of multiple molecular events and drug effects at molecular and cellular levels. Therefore, molecular imaging has been widely applied to assess disease progression at the molecular pathologic level for early diagnosis of cancer as well as neurological and cardiovascular diseases. Hence, the development of a molecular imaging probe to detect EGFRvIII expression before radiotherapy or chemotherapy would enable more accurate patient prognosis and prediction of drug sensitivity.

In this study, we developed a nuclear molecular imaging probe by labeling a novel anti-EGFRvIII mAb, 4G1, with a radioisotope and evaluated its potential to detect EGFRvIII expression in glioblastoma xenograft models by single-photon emission computed tomography (SPECT) imaging.

## RESULTS

### Production and characterization of novel mAb against EGFRvIII

After fusion of SP2/0 myeloma cells and spleen cells from immunized BALB/c mice, 157 positive hybridoma clones were obtained after initial ELISA screening. Among them, four hybridoma clones with the highest titer (4G1, 1F1, 7C7 and 4D3) were selected for further expansion after repeated screening. Finally, 4G1 was selected for further study because it had the highest titer, which immunoglobulin subtype was IgG2a.

### Affinity and specificity of 4G1

**Figure 1:**
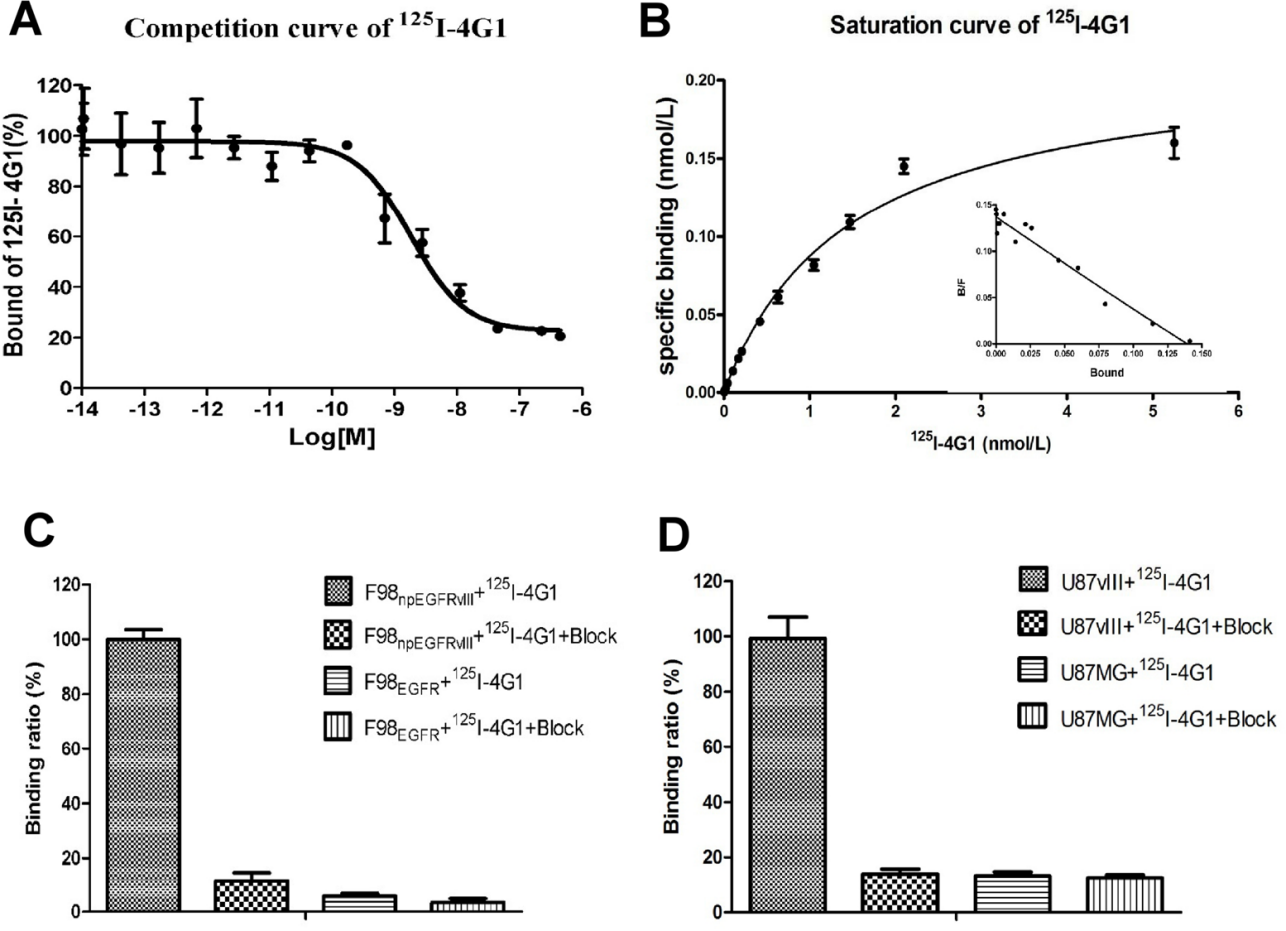
*In vitro* inhibition of ^125^I-4G1 binding to EGFRvIII on F98_npEGFRvIII_ cells by unlabeled 4G1 showed that the IC_50_ value was 1.83 ± 0.03 nmol/L (*n* = 3, mean ± SD) (**A**). Saturation binding of ^125^I-4G1 to EGFRvIII on F98_npEGFRvIII_ cells showed that the Kd value was 4.83 ± 0.12 nmol/L. Bmax was calculated to be approximately 1.21 ± 0.61 × 10^6^ sites/cell (**B**). Cell binding assays showed that ^125^I-4G1 specifically bound to F98_npEGFRvIII_ and U87vIII cells, but not F98_npEGFR_ and U87MG cells that express wild-type EGFR (**C**, **D**).

Several experiments were performed to evaluate the affinity and specificity of 4G1. As shown in Figure 1A, the IC_50_ value of ^125^I-4G1 was 1.83 ± 0.03 nmol/L. To determine the Kd of ^125^I-4G1 and number of binding sites per F98_npEGFRvIII_ cell (Bmax), we performed a saturation binding assay. The Kd value was 4.83 ± 0.12 nmol/L, and the Bmax was approximately 1.21 ± 0.61 × 10^6^ sites/cell (Figure 1B).

**Figure 2:**
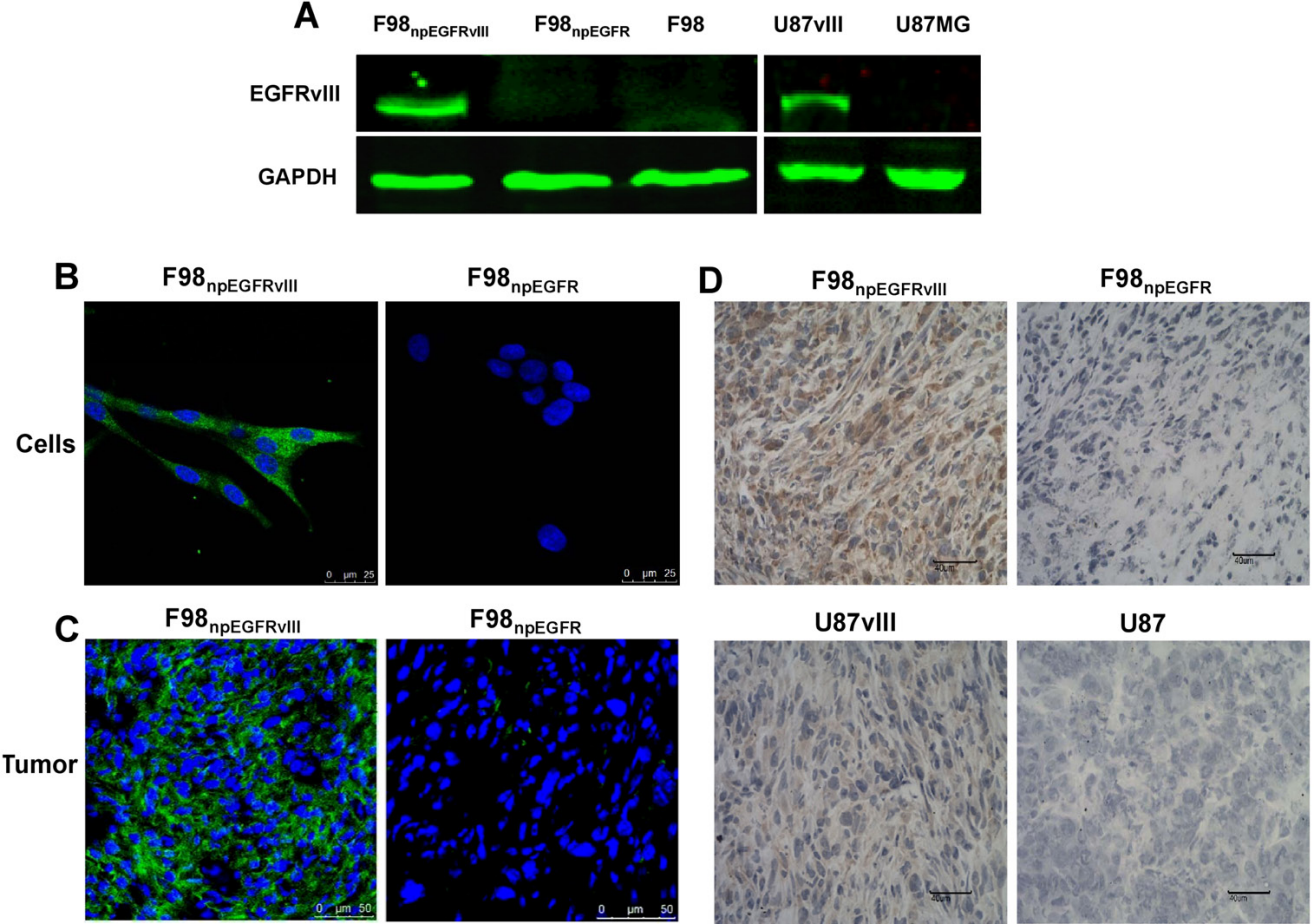
Western blot results showed that 4G1 exclusively recognized EGFRvIII protein over-expressed by F98npEGFRvIII and U87vIII cells (**A**) Immunofluorescence verified the specificity of 4G1 to F98_npEGFRvIII_ cells and xenografted tumors (**B**, **C**) Immunohistochemistry verified the specificity of 4G1 to F98_npEGFRvIII_ and U87vIII xenografted tumors (**D**).

**Figure 3:**
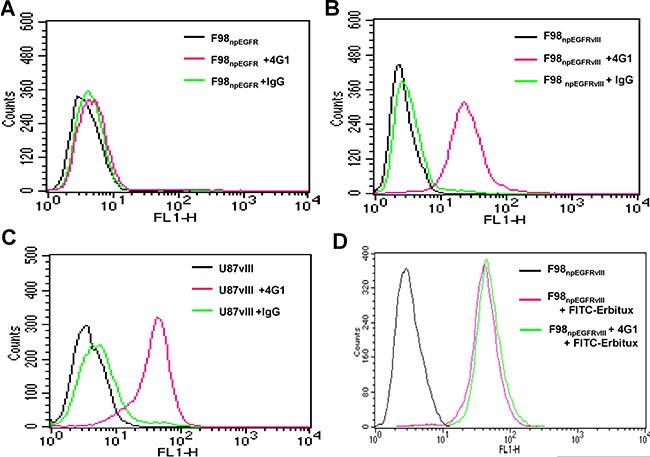
The positive rates of F98_npEGFRvIII_ (**B**) and U87vIII (**C**) cells stained with 4G1 were 92.5% and 83.4%, respectively, whereas 4G1 did not bind to F98_npEGFR_ and U87MG cells (**A**, C). The positive rate of F98_npEGFRvIII_ cells stained with Erbitux-FITC (1.0 μg) was 98.5% in the presence of excess 4G1 (200 μg) (**D**).

The binding assay results showed that ^125^I-4G1 exclusively bound to the EGFRvIII protein expressed by F98_npEGFRvIII_ and U87vIII cells, moreover unlabeled 4G1 blocked this specific binding (Figure 1C, 1D). The specificity was also confirmed by western blotting, immunofluorescence, and flow cytometric analysis. In western blot analyses, 4G1 exclusively recognized EGFRvIII expressed by F98_npEGFRvIII_ and U87vIII cells but not wild-type EGFR expressed by F98_npEGFR_ and U87MG cells (Figure 2A). Immunofluorescence and immunohistochemistry confirmed that 4G1 exclusively bound to EGFRvIII-positive cells and tumor tissues (Figure 2B–2D). Flow cytometry results showed that the positive rate of F98_npEGFRvIII_ and U87vIII cells stained with 4G1 was 92.5% and 83.4%, respectively (Figure 3B, 3C), whereas 4G1 did not bind to F98_npEGFR_ cells (Figure 3A). Furthermore, Flow cytometric analysis showed that 4G1 could not block the binding of Erbitux (a mAb against EGFR) to EGFRvIII on F98_npEGFRvIII_ cells, indicating that 4G1 had no common binding sites with Erbitux (Figure 3D).

### Biodistribution of ^125^I-4G1

**Figure 4:**
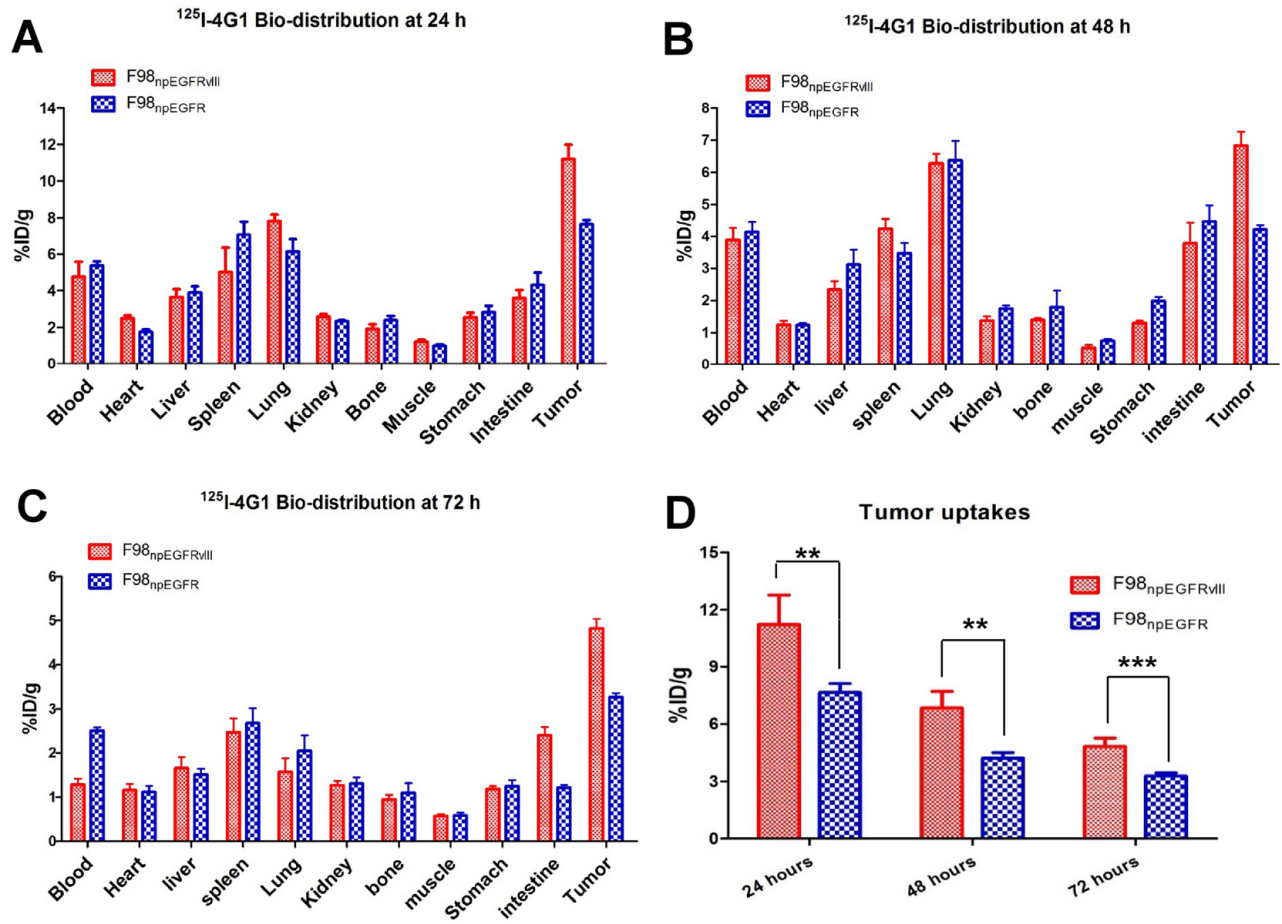
Biodistribution of ^125^I-4G1 in F98_npEGFRvIII_ and F98_npEGFR_ tumor-bearing nude mice at 24 h (**A**), 48 h (**B**), and 72 h (**C**) p.i. (**D**) ^125^I-4G1 uptake in F98_npEGFRvIII_ tumors was significantly higher than that in F98_npEGFR_ tumors. Data are expressed as the mean ± SD (*n* = 4 per group). **P < 0.01 and ***P < 0.001.

**Figure 5:**
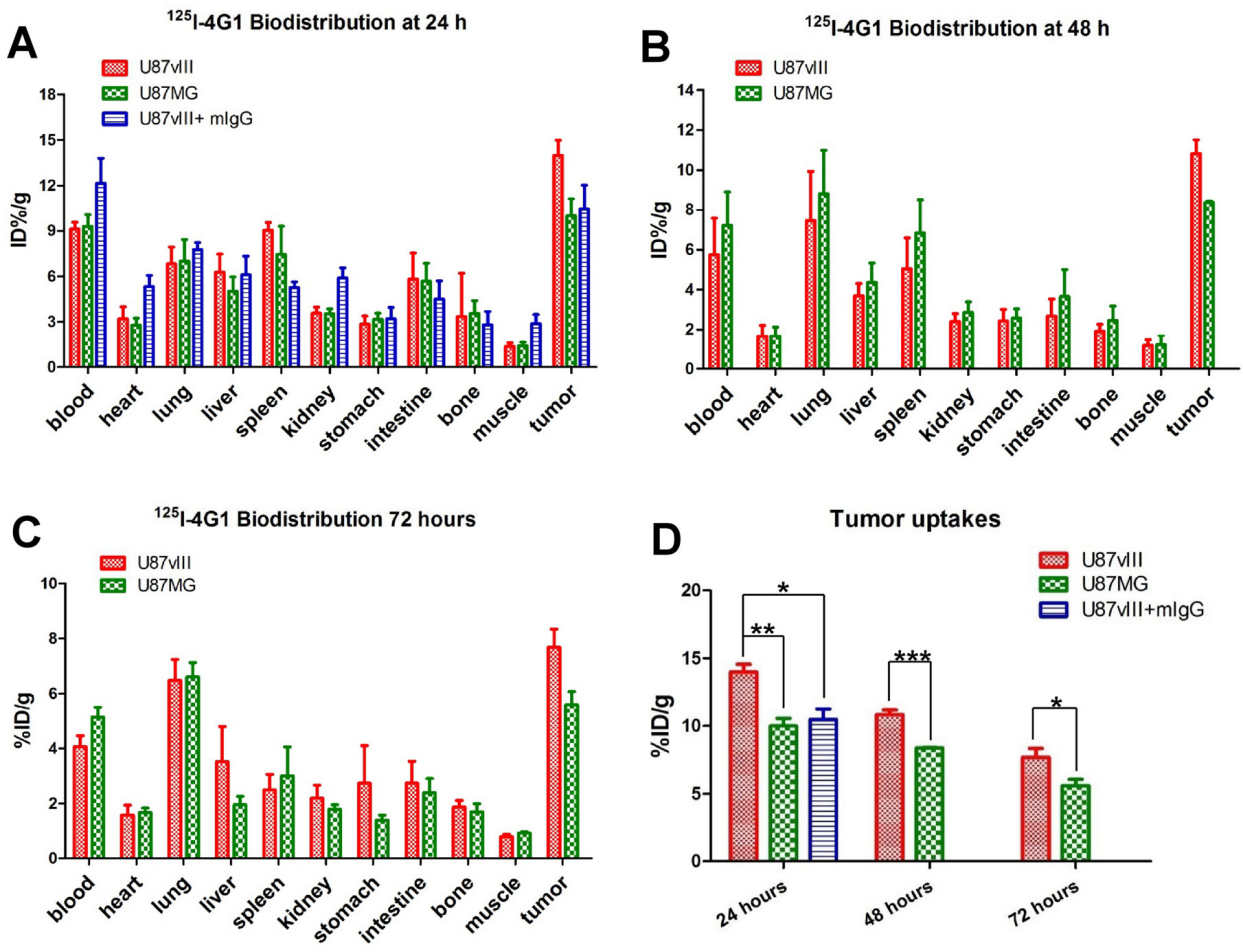
Biodistribution of ^125^I-4G1 in U87vIII and U87MG tumor-bearing nude mice at 24 h (**A**), 48 h (**B**) and 72 h (**C**) p.i. (**D**) ^125^I-4G1 uptake in U87vIII tumors was significantly higher than that in U87MG tumors. Data are expressed as the mean ± SD (*n* = 4 per group). *P < 0.05, **P < 0.01, and ***P < 0.001.

The biodistribution of ^125^I-4G1 was examined in F98_npEGFR_, F98_npEGFRvIII_, U87MG and U87vIII tumor-bearing BALB/c nude mice (Figures 4, 5). As shown in Figure 4D, uptake of ^125^I-4G1 in F98_npEGFRvIII_ tumors was 11.20 ± 0.75%ID/g, 6.82 ± 0.44%ID/g, 4.82 ± 0.22%ID/g at 24, 48 and 72 h p.i., respectively. These values were all significantly higher than ^125^I-4G1 uptake by F98_npEGFR_ tumors at the corresponding time points (7.64 ± 0.24%ID/g, 4.05 ± 0.30%ID/g, and 3.27 ± 0.09%ID/g, respectively).

Elimination of blood radioactivity in mice with F98_npEGFRvIII_ and F98_npEGFR_ tumors ranged from 4.75 ± 0.81%ID/g and 4.38 ± 0.22%ID/g at 24 h p.i. to 1.28 ± 0.13%ID/g and 2.51 ± 0.08 %ID/g at 72 h p.i., respectively. For most organs, there was progressive washout of radioactivity over time, which was concomitant with the clearance of radioactivity from the blood. The uptake of ^125^I-4G1 in all organs was almost less than 3%ID/g at day 3 p.i.

The *in vivo* EGFRvIII-targeting specificity of ^125^I-4G1 was confirmed by examining the biodistribution of ^125^I-mIgG in U87vIII tumor-bearing nude mice. As shown in Figure 5D, the tumor uptake of ^125^I-4G1 was significantly higher than that of ^125^I-mIgG (14.02 ± 0.40%ID/g vs. 10.46 ± 0.78%ID/g, *P* = 0.0069) at 24 h p.i. As shown in Figure 5, U87vIII tumor uptake of ^125^I-4G1 at 24, 48 and 72 h p.i. was 13.98 ± 0.57%ID/g, 10.82 ± 0.34%ID/g, and 7.68 ± 0.66%ID/g, respectively. These values were significantly higher than the U87MG tumor uptake of ^125^I-4G1 (9.98 ± 0.57%ID/g, 8.37 ± 0.03%ID/g, and 5.59 ± 0.47%ID/g, at 24, 48 and 72 h p.i., respectively).

### SPECT/CT Imaging of ^125^I-4G1

**Figure 6:**
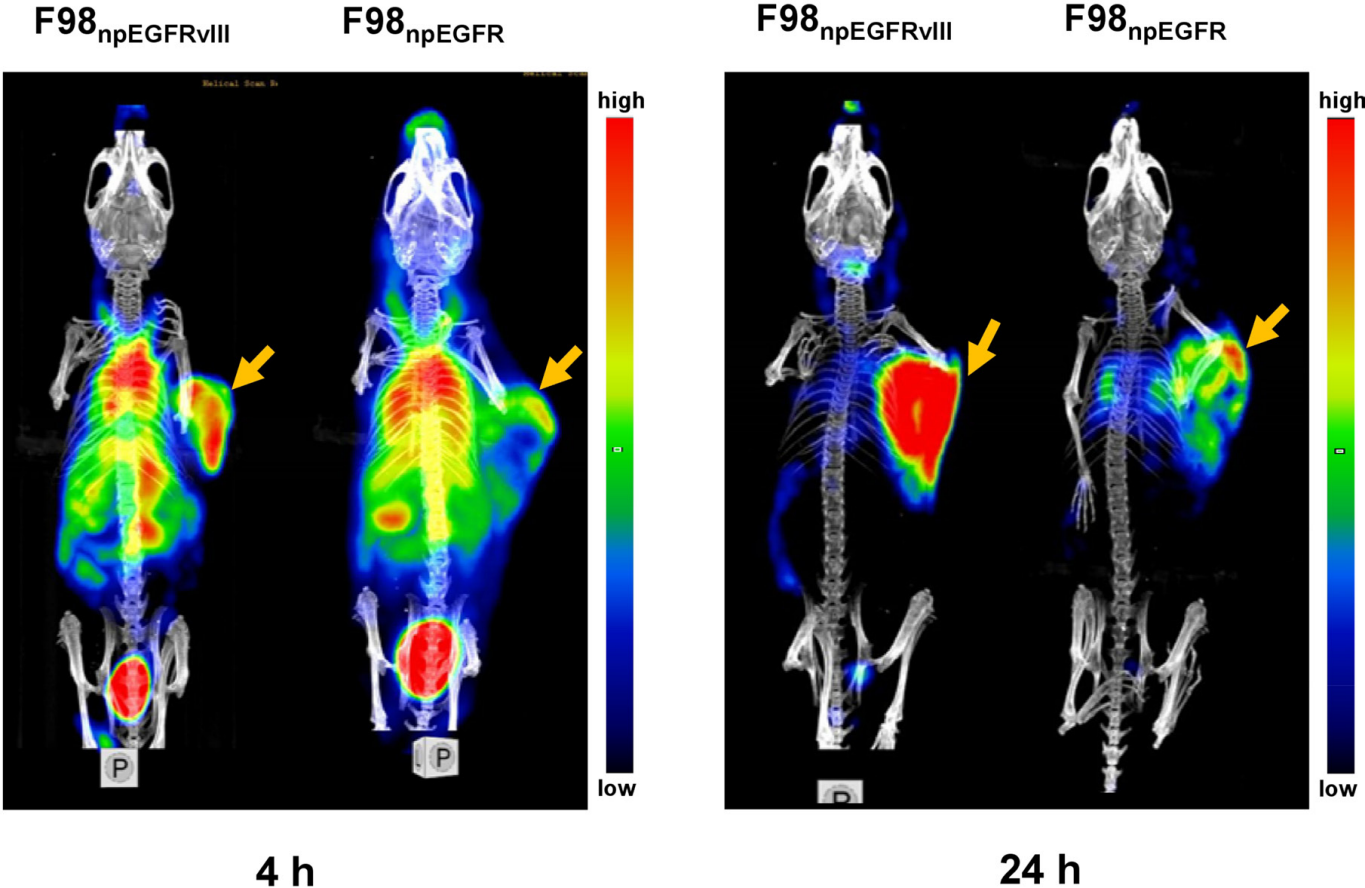
Representative small animal SPECT/CT images of F98npEGFRvIII and F98npEGFR tumor-bearing mice at 4 and 24 h p.i. 125I-4G1 uptake in F98npEGFRvIII tumors was significantly higher than that in F98npEGFR tumors at each time point The arrows indicate the F98_npEGFRvIII_ and F98_npEGFR_ subcutaneous tumors.

**Figure 7:**
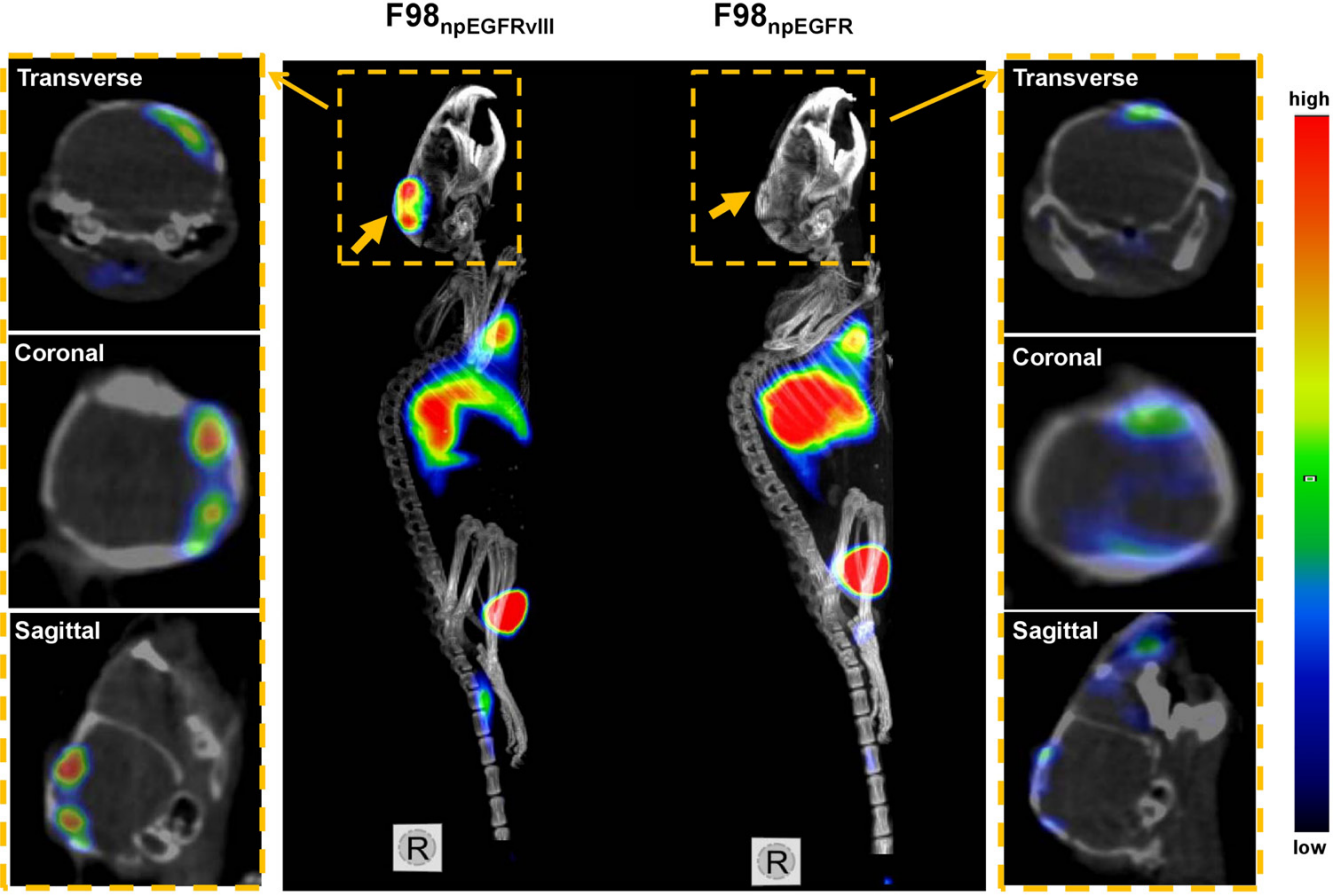
Representative small animal SPECT/CT images of F98npEGFRvIII and F98npEGFR intracranial tumor models at 24 h p.i. 125I-4G1 uptake in F98npEGFRvIII tumors was significantly higher than that in F98npEGFR tumors . The middle panel is MIP *(Maximum Intensity Projection)* images of F98_npEGFRvIII_ and F98_npEGFR_ tumors *in situ*, and the both side panels are the corresponding transverse, coronal and sagittal tomography images. The wide arrows in MIP images indicate the tumors *in situ*, while the narrow arrows indicate the tomography images of each tumor from the MIP images.

Representative SPECT/CT images of F98_npEGFR_ and F98_npEGFRvIII_ tumor-bearing BALB/c nude mice are shown in Figure 6. Based on the imaging results, the uptake of ^125^I-4G1 in F98_npEGFRvIII_ tumors was significantly higher than that in F98_npEGFR_ tumors at each time point. A small amount of radioactivity was also found in blood-rich organs, such as lung and heart, as well as liver at 4 h p.i. The background radioactivity decreased over time, which was consistent with the *ex vivo* biodistribution data. Images with a higher contrast were obtained at 24 h p.i. Moreover, the SPECT/CT images of F98_npEGFR_ and F98_npEGFRvIII_ intracranial tumor models as shown in Figure 7 showed that ^125^I-4G1 was markedly higher accumulated in EGFRvIII positive glioma than EGFRvIII negative glioma, which indicates that 4G1 might be used for real-time imaging studies in patients with gliomas in future.

## DISCUSSION

Preclinical investigations have shown that EGFRvIII stimulates unregulated growth, survival and invasion of glioma cells, as well as angiogenesis [26, 27]. In addition, the prognostic value of EGFRvIII has been studied extensively. Current viewpoints support the notion that the presence of EGFRvIII is an independent and significant unfavorable prognostic indicator of survival [16, 17, 28, 29]. Furthermore, EGFRvIII has been implicated in resistance to chemotherapy and radiotherapy [19–21]. Therefore, early detection of EGFRvIII expression might provide an early prognosis and suggest chemotherapeutic drug resistance. There have been several reports of EGFRvIII detection, but their results remain controversial, partly because of the lack of a consistently used EGFRvIII-specific antibody and detection assays. Moreover, these methods can not be applied to *in vivo* and real-time detection of EGFRvIII. Hence, a more sensitive, accurate and effective detection method should be developed and implemented.

To address this issue, we adopted a recently developed research methodology, molecular imaging, which enables noninvasive *in vivo* imaging and real-time monitoring of molecular biological processes at cellular and sub-cellular levels. Thus far, molecular imaging has been successfully applied to individualized therapies by assessment of disease progression, drug resistance, and disease prognosis [30–32].

In this study, we developed a novel mAb, named 4G1, to detect EGFRvIII expression through molecular imaging. The affinity assay results indicated that affinity of 4G1 to EGFRvIII was as good as other similar mAbs reported by previous studies [33, 34]. A series of specificity tests showed that 4G1 specifically recognized EGFRvIII and had no cross reactivity with wild-type EGFR. These results indicated that 4G1 had the potential to be a suitable targeting molecule for EGFRvIII-positive tumor imaging. To confirm this potential, we performed biodistribution and SPECT/CT imaging in xenografted tumor models. The results of biodistribution showed that EGFRvIII-positive tumors had a higher uptake of ^125^I-4G1 than EGFRvIII-negative tumors. Furtherly, in the biodistribution experiment of U87vIII tumor-bearing mice, a group of ^125^I-mIgG was done to confirm the specific uptake of ^125^I-4G1 in tumors. Since mIgG could not bind to EGFRvIII, the accumulation of ^125^I-mIgG in U87vIII tumors should be lower than that of ^125^I-4G1 and thus regarded as the background attributed to tumor non-specific uptake of antibody.

Furthermore, SPECT/CT imaging of ^125^I-4G1 showed that EGFRvIII-positive and -negative tumors could be distinguished easily. Thus, radiolabeled 4G1 may be a promising probe targeting EGFRvIII for tumor imaging.

We also examined potential common binding sites for 4G1 and Erbitux. Erbitux has been granted full approval by the U.S. Food and Drug Administration for the first-line treatment of patients with EGFR-expressing metastatic colorectal cancer [35]. Numerous studies have shown that Erbitux recognizes both EGFR and EGFRvIII [36, 37]. Our flow cytometric analyses revealed that 4G1 had no common binding sites with Erbitux. We also confirmed high affinity, specificity, and *in vivo* tumor accumulation of the 4G1 mAb. Considering that EGFRvIII is a prognostic and chemo/radio resistant marker, 4G1 can be easily labeled with PET or SPECT radioisotopes for potential clinical imaging of EGFRvIII expression. Moreover, 4G1 can be used as a delivery vehicle for tumor-targeted therapeutics, because EGFRvIII is exclusively expressed in tumor tissues.

Since EGFRvIII is a very promising therapeutic and diagnostic target that is exclusively expressed in tumors, it has attracted much attention. Over the past 10 years, some polyclonal [38–40] or monoclonal antibodies against EGFRvIII have been developed. In consideration of potential immunological cross reaction or difference among batches of polyclonal antibody, we excluded it in the first place. For monoclonal antibodies against EGFRvIII, the mAb 806 [41] and CH12 [33] were generated by immunization of mice with EGFRvIII-overexpressing fibroblasts, whereas L8A4 [42–44] was prepared by immunization of mice with peptides that were very similar to the peptides used in this study. These three mAbs have been evaluated in detail and applied to a range of research fields including EGFRvIII-xenograft therapy, immune-detection in pathological sections, drug delivery, and synergic treatment. In addition, mAb D2C7 [45], Erbitux and mAb 528 [46] have been confirmed to bind to both wild-type EGFR and EGFRvIII, which partly limits their applications and research value. To the best of our knowledge, no antibody has been applied to nuclear medical imaging to observe EGFRvIII expression *in vivo* so far. Thus, our study provides the first systematic evaluation of immunoSPECT imaging using an isotope-labeled EGFRvIII mAb (^125^I-4G1) to detect EGFRvIII expression, which provides a new real-time approach for the detection of EGFRvIII.

## MATERIALS AND METHODS

### Cell culture, plasmids, and peptides

U87MG human glioblastoma and F98, F98_npEGFR_, and F98_npEGFRvIII_ rat glioblastoma cell lines were purchased from the American Type Culture Collection (Rockvill, MD, USA). U87vIII cells were derived from U87MG cells stably transfected with the pEGFRP-N1-EGFRvIII plasmid (a gift from Dr. Janusz Rak, Cancer and Angiogenesis Laboratory, MUHCRI-Montreal Children's Hospital Research Institute, Canada) and modified to overexpress EGFRvIII protein. U87MG and F98 cells were cultured in low or high Glucose Dulbecco's modified Eagle's medium (DMEM) supplemented with 10% fetal bovine serum (FBS) at 37°C with 5% CO_2_. F98_npEGFR_, F98_npEGFRvIII_, and U87vIII cells were cultured in medium with 0.2 mg/mL G-418. Three EGFRvIII peptides (LEEKKGNYVVTDHC, EKKGNYVVTDHC, and KKGNYVVTDHC) were synthesized by GL Biochem Co., Ltd, (Shanghai, China). These peptides were conjugated with keyhole limpet hemocyanin (KLH) and mixed at a ratio of 1:2:3. For enzyme-linked immunosorbent assays (ELISAs), these three peptides were conjugated with bovine serum albumin (BSA) as a coating antigen.

### Antibody preparation

A series of anti-EGFRvIII mAbs were prepared by AbMax Biotechnology Co., Ltd, (Beijing, China). Briefly, three BALB/c mice were immunized subcutaneously three times with the above three peptides at 2-week intervals. The titer of antibodies against EGFRvIII in blood samples was determined by ELISAs using the three peptide-BSA conjugates or F98_npEGFRvIII_ cells as the coating antigens. Next, mouse spleen cells were collected and fused with SP2/0 myeloma cells. The fused cells were cultured in 10% FBS/DMEM containing hypoxanthine-aminopterin-thymidine (Sigma, St. Louis, MO, USA). Hybridoma colonies were maintained in 10% FBS/DMEM containing hypoxanthine-thymidine (Sigma). Hybridoma supernatants were repeatedly screened by ELISA. Finally, ascites were prepared by intraperitoneal injection of mice with 2 × 10^6^ hybridoma cells. Ascites titers were also determined by ELISA. Furthermore, analysis of the mAb subtype was performed with standard procedures provided by the protocol of the SBA Clonotyping™ System/HRP (Southern Biotechnology Associates, Inc., Birmingham, UK). The mAb with highest titer was named 4G1.

### *In Vitro* affinity and specificity evaluation of 4G1 mAb

### Affinity determination

For ^125^I radiolabeling, 10 μg 4G1 and 37 MBq Na^125^I (Beijing Atom High Tech, Beijing, China) in 0.2 M (pH 7.4) phosphate-buffered (PB) were added to a vial coated with 40 μg iodogen (Sigma). After 7 min of reacting at room temperature, the reaction mixture was purified by a PD-10 column (Amersham, Piscataway, NJ, USA).

The binding affinity of ^125^I-4G1 for EGFRvIII was evaluated by a saturation binding assay. Briefly, Increasing concentrations of ^125^I-4G1 (0–15 nmol/L) were added to F98_npEGFRvIII_ cells. The total volume was adjusted to 300 μL. Nonspecific binding was determined in the presence of an excess (> 500-fold) of unlabeled 4G1. After incubation at 4°C for 4 h, the cells were washed three times with cold PBS and then solubilized with 2 mol/L NaOH. The cell-associated radioactivity was determined using a γ-counter (Wallac 1470–002, PerkinElmer, Finland). A saturation binding curve and Scatchard transformation were obtained by nonlinear regression analysis, and the binding affinity (Kd) value of ^125^I-4G1 was determined using GraphPad Prism 4.0 (GraphPad Software, San Diego, CA). The experiment was performed twice and each data point represents the average value from triplicate wells.

The binding specificity of 4G1 to EGFRvIII was evaluated in F98_npEGFRvIII_ cells by the competitive binding assay. F98_npEGFRvIII_ cells seeded in a 48-well plate (5 × 10^4^ cells per well) were incubated at 37°C in a humidified atmosphere with 5% CO_2_ overnight to allow adherence. After washing with PBS, 3.7 kBq ^125^I-4G1 were added to each well in the presence of increasing concentrations of unlabeled 4G1. The cells were then incubated at 4°C for 4 h. After washed three times with cold PBS and solubilized with 2 mol/L NaOH, specific binding of ^125^I-4G1 to F98_npEGFRvIII_ cells was analyzed by measuring the radioactivity using the γ-counter. The experiment was performed twice with triplicate samples.

### Specificity determination

### Cell binding assay

F98_npEGFR_, F98_npEGFRvIII_, U87MG and U87vIII cells were trypsinized, and a single cell suspension of 1 × 10^6^ cells were prepared in 1 ml of 1% BSA/PBS (w/v). Then, 3.7 kBq ^125^I-4G1 was added to cell suspensions, followed by incubation at 4°C for 4 h. Nonspecific binding was determined in the presence of an excess (2 μg) of unlabeled 4G1. After washing with PBS, the specific binding of ^125^I-4G1 to cells was analyzed by measuring the radioactivity using γ-counter.

### Western blot analysis

Cells were harvested, washed three times with PBS, centrifuged, and then lysed. The protein concentrations of the lysates were determined using BCA Protein Assay Reagent (Pierce Chemical, Rockford, IL, USA). Total protein (50 μg) was separated by 10% sodium dodecyl sulfate-polyacrylamide gel electrophoresis and then transferred onto nitrocellulose membranes at 250 V for 2 h. Membranes were blocked for 1 h with 5% non-fat milk powder in TBS/0.1% Tween 20 and then incubated with 4G1 or rabbit anti-GAPDH polyclonal IgG (Cell Signaling Technology, Beverly, MA, USA) at 4°C overnight. The membranes were washed and incubated with a corresponding secondary antibody conjugated with Dylight 800 (EarthOx, San Francisco, CA, USA). After washing, immunoblotted proteins were detected using an Odyssey Western Blotting Detection System (Gene, Hong Kong, China).

### Immunofluorescence assay

Cells grown on LabTek chamber slides or frozen sections of tumor tissues were fixed with 2% paraformaldehyde/PBS (v/v) for 15 min on ice. After blocking for 30 min with 2% BSA and 1% normal goat serum, the cells or sections were incubated with 4G1 at 4°C overnight and then the corresponding fluorescently conjugated secondary antibody. Mouse IgG was used as an isotype control. DAPI was used for nuclear staining. Fluorescence signals were detected using a confocal microscope (TCS SP5; Leica, Germany). Immunohistochemistry was performed according to previous study [18].

### Flow cytometric analysis

Cells were trypsinized, and a single cell suspension of 1 × 10^6^ cells was prepared in 100 μL of 1% BSA/PBS (w/v) and immediately stained for 2 h at room temperature with relevant primary antibodies and then incubated with the corresponding fluorescently conjugated secondary antibodies. The primary antibodies included 4G1 and Erbitux-FITC. Human IgG (Becton-Dickinson, Rutherford, NJ, USA) was used as an isotype control. Stained cells were analyzed by a FACSCalibur flow cytometer (Becton-Dickinson, Rutherford, NJ, USA).

### *In Vivo* evaluation of 4G1

### Subcutaneous and orthotopic tumor models

All animal experiments were performed in accordance with the Guidelines of the Peking University Health Science Center Animal Care and Use Committee. For the subcutaneous tumor model, cells from each cell line (2 × 10^6^) in 100 μl PBS were injected into the right upper flanks of female BALB/c nude mice. When the tumor size reached 200–300 mm^3^, the animals were used for biodistribution and imaging studies. As for orthotopic brain tumor model, a total of 2 × 10^5^ cells of each cell line in 20 μl PBS were intracranially injected into the area between the cerebral cortex and dorsal hippocampus CA3 of experimental mice. When symptoms of hydrocephalus were observed, the mice immediately underwent SPECT/CT imaging [18].

### Biodistribution of ^125^I-4G1

F98_npEGFR_, F98_npEGFRvIII_, U87MG and U87vIII tumor-bearing nude mice were randomly divided into groups (*n* = 4 per group). Each mouse was injected intravenously with 185 kBq ^125^I-4G1. Mice were euthanized, and samples or organs of interest were removed, weighed, and analyzed in the γ-counter at 24, 48, and 72 h post-injection (p.i.). The results were calculated as a percentage of the injected dose per gram of tissue (%ID/g). To determine the specific accumulation of ^125^I-4G1 in U87vIII tumors, ^125^I-mIgG (185 kBq) was injected intravenously into four tumor-bearing mice, and then the biodistribution of ^125^I-mIgG at 24 h p.i. was determined as described above.

### Small animal SPECT/CT imaging

For small animal SPECT/CT imaging studies, each tumor-bearing nude mouse was injected intravenously with 55.5 MBq ^125^I-4G1 via the tail vein after anesthetization. The mice were placed in the prone position and imaged with a NanoScan (SPECT/CT) camera (Mediso Ltd, Budapest, Hungary) for 40 min. Imaging was performed at 4 and 24 h p.i. After completion of imaging, the mice were euthanized.

### Statistical analysis

Quantitative data are expressed as means ± SD. Means were compared using one-way analysis of variance (ANOVA) and Student’st test. *P* values < 0.05 were considered statistically significant.

## CONCLUSIONS

In summary, we generated an anti-EGFRvIII mAb, 4G1, which shows high receptor specificity, affinity and tumor uptake. Our results suggest that the radiolabeled 4G1 mAb is a promising probe to detect EGFRvIII expression *in vivo* by nuclear medicine imaging.
